# Drug control: initiatives from India

**DOI:** 10.1192/bji.2025.3

**Published:** 2025-05

**Authors:** Shalini Singh, Rakesh Chadda

**Affiliations:** 1 MD, DM, Associate Professor, National Drug Dependence Treatment Centre, All India Institute of Medical Sciences (AIIMS), New Delhi, India. Email: shalin.achra@gmail.com; 2 MD, Former Professor and Head, Department of Psychiatry and National Drug Dependence Treatment Centre, All India Institute of Medical Sciences (AIIMS), New Delhi, India

**Keywords:** Drug policy, substance use, illicit drugs, India, drug and narcotics control

## Abstract

The South Asian region, including India, faces an increased prevalence of illicit drug use. Key challenges include rising opioid use, injecting drug use and spread of stimulant use from some pockets to other regions of the country. Challenges faced are poor surveillance, lack of evidence-based and structured prevention programmes, wide treatment gaps and inadequate social capital for reintegration of substance users into society. The drug control efforts in India have resulted in an improved drug offence surveillance system, increased community awareness, a growing network of drug treatment centres and resource-building measures. India has made pioneering efforts in the field of harm reduction in the South Asian region. The steps taken have the potential of applicability across other South Asian, as well as most low- and middle-income, countries around the world.

Drug misuse is a major public health issue worldwide, and South Asia is no exception. India, with its massive population, faces a significant challenge in controlling illicit drug use. Cannabis and opioids are commonly used illicit drugs in India, with estimates suggesting that 22.6 million people currently use opioids, 31 million use cannabis and 2 million use stimulants.^1^ This article will focus on India's initiatives to curb illicit drug use. Figure 1 outlines the supply and demand reduction initiatives of various government agencies.

**Fig. 1:**
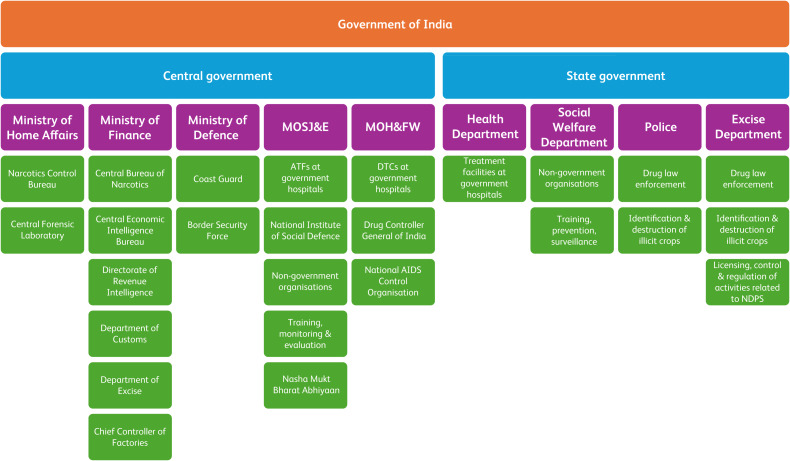
Division of responsibilities among various government bodies according to India's National Policy on Narcotic Drugs and Psychotropic Substances (NDPS), 2012. MOSJ&E, Ministry of Social Justice and Empowerment; MOH&FW, Ministry of Health and Family Welfare; ATFs, addiction treatment facilities; DTCs, drug treatment clinics.

## Challenges faced by India

A key challenge faced by India is the rapid increase in the prevalence of opioid use. The figure rose from 0.7% (12–60 age group) in 2004 to 3.97% (10–75 age group) in 2018, highlighting a concerning trend.^1^ The number of people who inject drugs has increased from about 200 000 to 850 000 in a decade. The treatment-seeking rate is around 25% in those suffering from dependence. The rates of in-patient treatment/hospital admission are even lower, at around 5%. The prevalence of pharmaceutical opioid use has also increased in the decade. Among children and adolescents, pharmaceutical opioids are a popular drug of choice for injecting drug use owing to easy availability and a perception that they are cheaper.^2^ Another worrying trend is the growing popularity of stimulants such as 3,4-methylenedioxymethamphetamine (MDMA, ecstasy), methamphetamine in the form of ‘crystal meth’ and *yaba* tablets (a mixture of methamphetamine and caffeine).^3^

## Initiatives by the Indian government

### Supply reduction

India's drug control policy has provisions for surveillance, prevention and management and penalties to empower the law enforcement agencies by criminalising the manufacture, trafficking, possession and personal consumption of illicit drugs under the Narcotic Drugs and Psychotropic Substances Act 1985 (NDPS Act). Table 1 outlines the penalties determined for contravention of the act. The Narcotics Control Bureau (NCB) is the primary enforcing body in the supply-reducing arm of drug control, with activities such as drug seizures, destruction of illicit drug cultivation areas, creation of the National Integrated Database on Arrested Narco Offenders (NIDAAN) Portal, which is a database of identification details of all individuals charged under the NDPS Act, and the National Narcotics Coordination (NCORD) Portal, which provides information on the source and destination of drugs (https://narcoordindia.gov.in/narcoordindia/index-english.php). According to recent a World Drug Report, the NCB has carried out sizeable seizure operations for reducing the global supply of heroin, opium poppy and cannabis.^4^ India's National Policy on Narcotic Drugs and Psychotropic Substances (NDPS), published in 2012, aligns with most of the supply reduction strategies of the NDPS Act. It has strategised how to tackle the recent rise in clandestine laboratories that are illegally manufacturing stimulants and newer psychoactive substances. The United Nations Office on Drugs and Crime (UNODC) and Ministry of Finance have carried out surveys in India to measure stimulant use and newer psychoactive substances respectively.^3,5^ The UNODC has organised capacity-building programmes to improve the law enforcement efforts in drug seizures.^6,7^

**Table 1:** Offences and penalties under India's Narcotic Drugs and Psychotropic Substances (NDPS) Act 1985 (amended in 2021)

	Production		Supply	Possession	Consumption
Is it an offence?	Yes		Yes	Yes	Yes
Is it a criminal offence?	Yes		Yes	Yes	Yes
Is there any immunity from prosecution?	Yes, for ‘addicts’ who are charged with offences involving small quantities of NDPS as defined by the act if they volunteer for treatment (section 64A) or are released on probation by the court if found suitable (section 39)		Yes, for ‘addicts’ who are charged with offences involving small quantities of NDPS as defined by the act if they volunteer for treatment (section 64A) or are released on probation by the court if found suitable (section 39)	Yes, for ‘addicts’ who are charged with offences involving small quantities of NDPS as defined by the act if they volunteer for treatment (section 64A) or are released on probation by the court if found suitable (section 39)	Yes, for ‘addicts’ who are charged with offences involving small quantities of NDPS as defined by the act if they volunteer for treatment (section 64A) or are released on probation by he court if found suitable (section 39)
Is there any exception for medical and scientific use?	Yes		Yes	Yes	Yes
Is the penalty based on quantity of drug?	Yes, except for coca leaf and coca plant (section 16)		Yes, except for coca leaf and coca plant (section 16)	Yes, except for coca leaf and coca plant (section 16)	No
Is the penalty based on type of drug?	Yes, in case of contraventions related to coca leaf and coca plant, the penalty is rigorous imprisonment^1^ for up to 10 years or a fine up to US$1250^2^ irrespective of the quantity (section 16)		Yes	Yes	Yes, the penalty is different for scheduled narcotic drugs and psychotropic substances and those not listed in the Official Gazette (section 27b)
Minimum penalty? (sections 15–27)	Small quantity	Rigorous imprisonment or fine (duration and amount not specified)	Rigorous imprisonment or fine (duration and amount not specified)	Rigorous imprisonment or fine (duration and amount not specified)	Rigorous imprisonment or fine (duration and amount not specified)
	Less than commercial quantity	Rigorous imprisonment or fine (duration and amount not specified)	Rigorous imprisonment or fine (duration and amount not specified)	Rigorous imprisonment or fine (duration and amount not specified)	
	Commercial quantity	10 years’ rigorous imprisonment and US$1250 fine	10 years’ rigorous imprisonment and US$1250 fine	10 years’ rigorous imprisonment and US$1250 fine	
Maximum penalty? (sections 15–27)	Small quantity	1-year rigorous imprisonment and US$125 fine	1-year rigorous imprisonment and US$125 fine	1-year rigorous imprisonment and US$125 fine	1-year rigorous imprisonment and US$125 fine
	Less than commercial quantity	10 years' rigorous imprisonment and US$1250 fine	10 years' rigorous imprisonment and US$1250 fine	10 years' rigorous imprisonment and US$1250 fine	
	Commercial quantity	20 years' rigorous imprisonment and US$2500 fine	20 years' rigorous imprisonment and US$2500 fine	20 years' rigorous imprisonment and US$2500 fine	
Enhanced punishment upon reconviction?	Yes (section 31)		Yes (section 31)	Yes (section 31)	Yes (section 31)
Death penalty?	Yes (section 31a)		Yes (section 31a)	Yes (section 31a)	No

1 Rigorous imprisonment is defined as hard labour in the Indian Penal Code.

2 Amounts are approximate in US$. In 2023 India's GDP per capita was 2481 USD.

### Demand and harm reduction

A shift towards prevention programmes that focus on age-specific life skills development, social cohesion and prosocial lifestyles at the individual, family and community level are crucial for demand reduction. To achieve this, the NAVCHETNA programme has been designed by the Ministry of Social Justice and Empowerment.^8^ India's demand reduction initiatives kickstarted with the Scheme for Prevention of Alcoholism and Substance (Drugs) Abuse in 1985–1986. The key focus of this scheme was prevention and rehabilitation and a key development was the setting up of integrated rehabilitation centres for addicts (IRCAs).^9^ The Drug De-addiction Programme was set up in 1988 to build a nationwide network of de-addiction clinics with the primary focus being provision of medical treatment for substance use disorders and capacity building. These schemes were partially successful.^10^ In 2014, the Ministry of Health and Family Welfare began to set up drug treatment clinics (DTCs) with the goal of providing only out-patient services. Such clinics have been opened in 27 hospitals around the country. In 2019, the Ministry of Social Justice and Empowerment launched the National Action Plan for Drug Demand Reduction (NAPDDR) to provide ‘financial assistance for preventive education and awareness generation, capacity building, skill development, vocational training and livelihood support of ex-drug addicts, programmes for drug demand reduction and running and maintenance of IRCAs’.^9^ Under this plan a nationwide network of addiction treatment facilities (ATFs) is being set up with a focus on both in-patient and out-patient care and availability of opioid substitution therapy. Thus far, 46 such facilities have been set up and the scale-up is ongoing. Community mobilisation is another area that needs focus. The Nasha Mukt Bharat Abhiyaan (‘Making the Country Free of Addiction’) campaign was launched in 2020 as part of the NAPDDR to decentralise the prevention, surveillance and needs assessment campaigns against substance use to district level. This integration of ATFs and DTCs into the existing healthcare systems is a best practice example of a demand reduction initiative in South Asia. As of 2022, most of the first-line medications used to treat drug dependence have been included in India's National List of Essential Medicines.

A key demand reduction objective of the NDPS policy is to strengthen training programmes and assist the voluntary sector and state-run hospitals in delivering affordable and quality care. The policy includes steps to formulate minimum standard of care guidelines for de-addiction centres and to monitor compliance. The policy directs the National Drug Dependence Treatment Centre (Delhi) to train medical personnel in providing drug dependence treatment and the country's National Institute of Social Defence to assist voluntary organisations and local governing bodies in increasing prevention and awareness efforts. The policy focuses on gender-sensitive treatment and rehabilitative services. For people who inject drugs, the policy supports harm reduction strategies such as needle/syringe exchange facilities and opioid substitution therapy, albeit for minimising the spread of blood-borne infections.

As a consequence this focus on harm reduction, one of India's significant contributions in the South Asian region has been its pioneering efforts in setting up widespread access to opioid substitution therapy, via the National AIDS Control Organization (NACO), and to the Needle Syringe Exchange Program (NSEP). A scale-up of opioid substitution therapy for all opioid-dependent patients has been planned under the Ministry of Health and Family Welfare's Drug De-addiction Programme,^10^ and one of the aims of India's National Strategic Plan for HIV/AIDS and STI for 2017–2024 is to increase accessibility to both opioid substitution therapy and the NSEP.

## Challenges faced by India in implementing these initiatives

Despite the initiatives taken by the Indian government, several challenges remain. The first set of these can be summarised as an unduly severe stance towards drug users Continued criminalisation of personal consumption indicates that drug use is primarily seen as a law-and-order problem and harm reduction measures have been viewed as a stopgap measure by policymakers. The NDPS policy lacks treatment, rehabilitation and harm reduction policies for people who inject drugs and those who are in prison. Measures to reduce drug use-related accidents and overdose do not feature in Indian policy. The policy has failed to outline clear strategies for targeting high prevalence of cannabis use and rising pharmaceutical opioid and stimulant use. Techniques to capture emerging drug use trends such as data mining and wastewater analysis do not yet form a part of drug control initiatives.

The second set of challenges is related specifically to populations served. Issues unique to special populations such as children, adolescents, migrants, prisoners, people who inject drugs and border area residents are not adequately addressed. India still lacks diverse treatment options for female drug users, adolescents and the lesbian, gay, bisexual, transgender and queer or questioning (LGBTQ) community. Implementation is another challenge. The prevention programmes are not being uniformly implemented across all educational institutions and no programme has been formulated for at-risk populations such as street children.^11^

Import and manufacturing quotas for narcotic drugs traditionally used in India, together with an ‘alternative development’ programme to re-educate farmers whose livelihoods have long depended on illicit crop cultivation, are part of policy. However, efforts to allow for regulated cultivation of low-risk traditional drug crops (such as opium poppy and cannabis for manufacture of afeem and bhaang) and ensure protection of the environment while eradicating illicit crops does not yet feature in Indian drug policy. Use of information technology as a tool for implementing supply control measures and training activities have been planned but safeguards are needed to protect sensitive data. India is yet to establish a monitoring and surveillance programme for measuring the extent and patterns of drug use, treatment-seeking rates and barriers to care.

## A comprehensive approach is needed

Addressing the underlying social, economic and political factors that contribute to drug use is critical. The government, civil society organisations, healthcare providers and community groups need to work closely together to achieve a sustainable impact through demand reduction programmes. The Indian government needs to scale up prevention and treatment oriented approaches. Currently, the treatment gap in India is significant, meaning many individuals who need treatment are unable to access it. To address this gap, the government needs to invest in training social workers, counsellors, educators and healthcare professionals and in creating more treatment facilities, including out-patient clinics, psychosocial services and residential centres. Experiences gained in India and the lessons learned have the potential of applicability to other South Asian countries and, indeed, to low- and middle-income countries around the world.

## Data Availability

Data availability is not applicable to this article as no new data were created or analysed in this study.
